# Hospital/clinical ethics committees' notion: an overview

**Published:** 2016-12-18

**Authors:** Fatemeh Hajibabaee, Soodabeh Joolaee, Mohammad Ali Cheraghi, Pooneh Salari, Patricia Rodney

**Affiliations:** 1 PhD Candidate in Nursing, Faculty of Nursing & Midwifery, Tehran University of Medical Sciences, Tehran, Iran;; 2 Associate Professor, Center for Nursing Care Research, Iran University of Medical Sciences,Tehran, Iran; Iranian Academy of Medical Sciences, Tehran, Iran;; 3 Associate Professor, School of Nursing & Midwifery, Tehran University of Medical Sciences, Tehran, Iran;; 4 Associate Professor, Medical Ethics and History of Medicine Research Center, Tehran University of Medical Sciences, Tehran, Iran;; 5 Associate Professor, School of Nursing, University of British Columbia, Vancouver, Canada.

**Keywords:** *Clinical ethics*, *Ethics committee*, *Ethics consultation*

## Abstract

Hospital ethics committees (HECs) help clinicians deal with the ethical challenges which have been raised during clinical practice. A comprehensive literature review was conducted to provide a historical background of the development of HECs internationally and describe their functions and practical challenges of their day to day work. This is the first part of a comprehensive literature review conducted between February 2014 and August 2016 by searching through scientific databases. The keyword ethics committee, combined with hospital, clinic, and institution, was used without a time limitation. All original and discussion articles, as well as other scientific documents were included. Of all the articles and theses found using these keywords, only 56 were consistent with the objectives of the study. Based on the review goals, the findings were divided into three main categories; the inception of HECs in the world, the function of HECs, and the challenges of HECs. According to the results, the Americas Region and European Region countries have been the most prominent considering the establishment of HECs. However, the majority of the Eastern Mediterranean Region and South-East Asia Region countries are only beginning to establish these committees in their hospitals. The results highlight the status and functions of HECs in different countries and may be used as a guide by health policymakers and managers who are at the inception of establishing these committees in their hospitals.

## Introduction

Owing to the new knowledge obtained from medical studies and the diversity of not only lifestyles, but also moral and religious values in modern societies, medical involvement and decision-making processes associated with healthcare are becoming more and more complicated. Moreover, noticeable economic pressure is being experienced by healthcare systems throughout the world, as awareness of ethical implications in medicine has been raised through public political and medico-legal discussions about patient independence, euthanasia and assisted dying (1). Csikai has noted that as complicated ethical issues have drawn widespread public attention and fulfilled the need to have protected patients' rights in the health care setting, the need for formal ethics committees has been recognized by most institutions (2). Much significance has been acquired by ethics committees in the hospital setting due to the developments in medical technology (2) around the late 20^th^ century. Over the past 30 years, having hospital ethics committees (HECs) has been encouraged or assigned to hospitals in many countries (3), whereas such committees have not been extensively present in the healthcare settings of developing countries (4).

Support has been provided to clinicians by HECs when they have met ethical challenges in their clinical practice (5, 6). These committees have made much progress in regards to doctors, nurses, and other health experts becoming acutely aware of the ethical decisions they have to make (4). A healthcare ethics committee or hospital ethics committee is characterized as a body of persons established by a hospital or health care institution and assigned to consider, debate, study, take action on, or report on ethical issues that arise in patient care (7). Over time, HECs have moved toward democratic processes of discussion by which ethical processes are advocated with the consideration of the benefit of patients, their families, and health care team members (6).

HECs have made considerable progress in many countries since the 1980s (8) and are now well established in many countries (9, 10).

An ethics committee consult is periodically called by nurses, to help patients and to get assistance in solving ethical dilemma. Preparation before asking for an ethics consultation is critically taken into account (5).

Ethical issues must be taken into consideration even more in nursing (11) and nurses should be educated on ethical issues through ongoing education programs, in-service programs, patient care conferences, and academic courses (12). Moreover, those who serve on an ethics committee have a personally and professionally rewarding experience due to the service provided for staff, patients, and families (5). The title “hospital ethics committee” can be utilized interchangeably with “patient care advisory committee”, "healthcare ethics committee", "clinical ethics committee", "institutional ethics committee", and "hospital clinical ethics committee" (3, 7). HECs are responsible for providing ethics consultations. They also evaluate the results of consultations when the consultant is an individual ethicist instead of a board of ethicists (13). Most published literature has reported policy review, education, and consultation as the leading roles of these committees (14). More and more, organizational ethical issues, such as the allocation of resources, are being addressed by the HECs (6). 

Ethics committees consist of members from many various disciplines in the health care setting. A holistic examination of a patient’s or their family’s situation that might involve a complicated ethical dilemma is possible through an interdisciplinary view of the issue (2). The various perspectives of nurses, chaplains, physicians, social workers, lawyers, and others brings variety to the debate and serves the patient in the best way possible (7). Committee members should be diverse in terms of their culture and skills, experiences, and knowledge. Variety inspires debates to obtain new information and consider alternative ideas (15), which is regarded as a prerequisite to culturally safe and ethically sound discussions (6). In order to prevent parochialism, a number of health professional members should be recruited from outside the institution (4). The committee members "should be inclined to learn about clinical ethics, receptive to various ideas and viewpoints, able to deal with emotionally charged topics and interpersonal disagreements, and capable of tolerating vagueness” (16). In general, the success of any HEC is dependent on the commitment and devotion of its members. As a matter of fact, there is increasing attention to the competencies needed for individuals engaged in ethics committees, especially ethics consultants (17).

Numerous studies have applied different methods to examine the performance and various dimensions of HECs in different countries in recent years, and have revealed similarities and differences. HECs still do not exist or have not been appropriately established in some developing countries, and few studies have been performed in this regard in these countries. Thus, it appears necessary to conduct studies that can provide guidelines for the establishment and operation of HECs through presenting an integrated comprehensive image of the process of development and performance of such committees. 

The three main goals of this review included to describe the history and development of HECs in the world [countries classified according to World Health Organization (WHO) Regional Offices], explain the function of HECs, and to discuss the challenges of their practice.

## Method

Initially, world literature was reviewed, and then, analyzed and compared to formulate an appropriate guideline to establish committees with favorable structure and performance in countries where the establishment of ethics committees in hospitals has been newly planned. Consequently, this is the first part of a comprehensive literature review conducted between February 2014 and August 2016 by searching through scientific databases such as PubMed, Ovid, Scopus, Web of Science, and Google Scholar. The keyword ethics committee was used in combination with hospital, clinic, and institution. All original and discussion articles, as well as other scientific documents were included without a time limitation. 

In the present study, countries with published studies on the history and procedures related to HECs development in English were included. The countries were classified according to WHO Regional Offices. This study was derived from the first author’s PhD thesis performed in Iran where HECs have lately been developed. 

## Results

Among all the articles and theses which were found with these keywords, only 56 were consistent with the objectives of this study. There were 5 theses, 18 quantitative studies, 3 qualitative studies, 21 discussion articles, 5 editorials, 1 commentary, 2 comparative studies, and 1 review study. Based on the ‎review goals, the findings were divided into three main categories; the history and development of HECs in the world, the function of HECs, and the challenges of HECs. 

**History and development of hospital ethics committees**

Of the documented literatures published on the history of HECs in different countries, 22 belong to western countries and began around 1985 (5, 7, 9, 13, 14, 18-33). The history and process of the development of HECs in western countries are provided in this section based on the year the committees were established.

*The United States*

Review committees were formed in the US following requests for the approval of all decisions about abortion in the 1960s (20). Due to the limited number of dialysis machines in the 1970s, a number of committees were developed to determine patients’ priority in access to hemodialysis (20, 21). Since HECs (also known as heath care ethics committees) were developed in the 1980s in the US, most available data on these committees are obtained from the American literature (5, 22). While only 1% of hospitals in the US had HECs in 1983, this rate increased to over 90% by 2001 (23). In 1984, the development of these committees was endorsed by the American Medical Association and the American Hospital Association. Their tasks were considered to be encompassing several areas and guiding hospital policy, but the key and most groundbreaking goal was to make recommendations in individual cases (34). There has been dynamic discussion over how valuable these committees truly are (35). Today, HECs are the primary mechanisms for managing ethical issues in clinical care in the USA (36, 37).

*Canada*

Limited HECs existed in Canada before the 1980s (13, 18). In 1986, the Guide to Accreditation of Health Care Facilities indicated the need for multidisciplinary ethics committees for the resolution of biomedical issues (38). Two national surveys of Canadian HECs in 1984 and 1989 reported that, respectively, 18% and 58% of the hospitals in the country had a HEC (18, 39). Today, however, the exact conditions of HECs in Canada are not known (13). Moreover, a study in 2010 found that 85% of the surveyed Canadian hospitals had HECs (13).

**European Region**

*Netherlands*

In the Netherlands, HECs were established in the early 1970s. For almost 20 years, however, they were mostly mixed committees. They started as bottom-up enterprises and have maintained their nature ever since (24). It has also been reported that ethics advice on clinical issues can be provided by HECs in the Netherlands (40).

*France*

The growth of bioethics and ethics committees in France differs greatly from that in other countries (25). Two major movements in France led to the formation of ethics committees in the 1980s. The first was a political movement which resulted in the development of a permanent national ethics committee called “*Comité Consultatif National d’Ethique*” (CCNE). The second, a professional movement, facilitated the formation of local ethics committees. During 1983-93, the CCNE focused on three major topics including medically assisted procreation and the embryo, research on human beings, and genetics (25).

*United Kingdom*

Among the European countries, United Kingdom was one of the pioneers in this regard. A number of local reasons, including institutional response to particular problematic cases and clinicians’ concern about the ethical aspects of clinical practice, were involved in the development of the first clinical ethics committees in the UK (22). One of the first HECs was created at what is now called Barts and The London NHS Trust, incorporating the formerly independent St. Bartholomew's and The Royal London Hospitals, The London Chest Hospital, and the Queen Elizabeth Hospital for Children. This HEC, which began its work in 1995, is now an integral feature of professional life within the trust and is a sub-committee of the trust's Clinical Governance Committee, working closely with the Medical Director. The terms of reference of the HEC emphasize two important dimensions (41). First, the committee acts proactively in the development of coherent and practical ethico-legal policies and provision of recommendations for the traditional issues commonly faced in clinical settings and mentioned in the literature related to medical ethics and law. The HECs’ second function is reactive and involves the provision of relevant advices on particular ethico-legal issues discussed by clinical colleagues (42).

*Slovakia*

 In Slovakia, in June 1992, the ‘‘Guidelines on Establishment and Work of Ethics Committees in Health Care Facilities and Biomedical Research Institutions’’ (43) were elaborated by the Central Ethics Committee, and published in the form of the Ministry of Health's recommendations. The guidelines provided detailed directions regarding the formation and responsibilities of local ethics committees (24).

*Belgium*

A law passed in 1994 obliged all general and psychiatric hospitals in Belgium to develop a local ethics committee to handle the responsibilities of both Research Ethics Committees (RECs) and HECs. This seems to be the origin of ethics committees in charge of both research and clinical ethics (26). In 2000, however, the Belgian Court of Arbitration excluded ethics consultation from the HEC-related tasks. Since the Belgian HECs are controlled on a national scale, all hospitals have their own ethics committee responsible for a large number of committee tasks (43).

*Norway*

In 1996, a group of hospital clinicians, politicians, and health authorities, along with the Norwegian Medical Association launched an initiative in Norway which led to the development of the first HECs in the country. As a result, there is at least one ethics committee at any of the 23 hospital trusts providing 4.9 million Norwegians with specialized and hospital-based healthcare services (44).

*Germany*

The establishment of a HEC is mandatory in all hospitals registered in the Christian Association of Hospitals in Germany (22). According to a new study in Germany, the percentage of hospitals with HECs was found to be 86.4% (45). 

*Croatia*

Among European countries, Croatia achieved the legal requirements for the establishment of ethics committees around 1997. The 1997 "Law on the Health Protection" obliges all healthcare institutions in Croatia to create an ethics committee. Each committee should have 5 members including 2 individuals who are not involved in the field of medicine (28). In Croatia, a top-down approach was adopted for the development of ‘‘mixed’’ ethics committees, i.e., a combination of HECs and RECs, in healthcare institutions (24). Ethics committees were established in almost half (46%) of all healthcare institutions in the country (except drugstores and homecare provision centers) 6 years after the reinforcement of the Law on the Health Protection in Croatia (in 2006). Most of these committees (89%) consist of 3 medical professionals and 2 experts in other fields. Moreover, while 49% of the committees were mainly involved in the analysis of research protocols, a small percentage of them provided standing orders, professional guidelines, or other related documents (28).

*Lithuania*

In Lithuania, HECs, called medical ethics commissions, are established based on the Health Care System Law of the Republic of Lithuania and the Model Guidelines for Medical Ethics Commissions (released by the Ministry of Health in 1997). The Health Care System Law of the Republic of Lithuania was passed by the Parliament in the early 1990s and obliged all large healthcare institutions to create a specific HEC. The Lithuanian National Bioethics Committee was then established in response to the need for an organization to manage and support these HECs. The Model Guidelines for Medical Ethics Commissions provided details on the mission, functions, establishment, and composition of HECs (24).

*Turkey*

Although ethics committees exist in university hospitals in Turkey, so far, these committees have had no advantages over pharmaceutical RECs. Due to the need for HECs to resolve the ethical issues faced by not only doctors, but also patients, the senate of Kocaeli University Medical Faculty in Turkey approved the establishment of a HEC on November 13, 2000 (46). Demir and Buken stated: "In Turkey, The introduction of HECs is relatively recent and the number of committees is limited" (10).

*Poland*

There were no documents of existing HECs in Polish hospitals until 2007 (43). We could not find any legal or ethical regulation concerning HECs. Medical ethics committees of medical councils or chambers of physicians and dentists, working at national and regional levels, are the only committees in Poland which handle issues related to medical ethics and have similar functions (but not exactly) to those of HECs. However, they have limited effects on the development of healthcare policies and clinical decision-making. In 1990, the first General Medical Assembly, the highest authority of the Polish Chamber of Physicians and Dentists (PCHPD), recommended the establishment of the Medical Ethics Committee of the Supreme Medical Council (43). Nevertheless, not many hospitals in Poland currently have HECs and the existing committees usually fail to provide the required structure, services, and workload (29).

**Western Pacific Region**

*Australia*

Despite their ongoing development in Australia, HECs and other ethics support services are generally not accessible by all institutions (47). The Committee at John Hunter has published their experience (48), but there are not many documents about Australian HECs. In New South Wales and other parts of Australia, public hospitals are supposed to focus on providing clinical ethics support as a priority (49).

*China*

Chinese Medical Association (CMA) set up the HEC as one of its branches in 1988. The Regulation of Hospital Ethical Committees in China was issued at the sixth conference of medical ethics in Chengdu in 1991, and then, revised by the CMA in 1995 (50). In 2007, the Ministry of Health of China published a review of methods used in biomedical research involving humans. Today, all medical institutions and hospitals in the country have an ethics committee whose main function is to ensure respect for principles of autonomy, beneficence, and justice (50).

*Japan*

In Japan, 20 (25.6%) medical organizations developed an ethics committee in 1998, and this rate increased to 29 (50.0%) in 2003 (50). A recent study on 4000 hospitals in Japan reported 51.1% of the surveyed hospitals to have an ethics committee. Moreover, 16.8% of the hospitals were working on developing their HECs (51).

*Korea*

When faced with ethical dilemmas in university hospitals, residents and physicians in Korea have very limited access to clinical ethicists or active hospital ethics committees to consult with (52). According to a study in Korea, only 3.4% of the surveyed residents had discussed their ethical issues with an attending faculty member or a hospital ethics committee. The participating residents tended to resolve their ethical conflicts on their own (15.1%) or by asking for advice from their colleagues or senior residents (44.9%) or the hospital’s ethics committee (0.7%) (52).

*New Zealand*

A total of 15 Health and Disability Ethics Committees have been established in New Zealand. These committees are currently working based on the national guidelines (53). These committees are accredited by the Health Research Council Ethics Committee for the ethical review and approval of research (4).

The Report of the Inquiry into National Women’s Hospital (1988) concluded that the ethical review of research proposals and other issues should be performed by independent ethics committees consisting of 50% lay people (not involved in health professions) and also chaired by a lay person (53). 

**Eastern Mediterranean Region**

*Iran*

In the previous two decades, there have been remarkable movements in the field of bio-medical ethics in Iran, mainly in the educational, research, and policy-making aspects (54). The regulations and guidelines for the HECs were established by the Iranian Ministry of Health and Medical Sciences in 2000. Based on a national study for identifying the priorities of medical ethics in Iran, the constitution of HECs was one of 10 priorities (55); however, HECs are not yet working as strong as other hospital committees. The results of a study conducted in one of the major cities in Iran indicates that HECs are functioning as combined committees (85.7%) entitled ethics committee and religious principles. These committees are chaired by doctors (42.8%), hospital directors (2.5%), hospital managers (14.28%), and nurses (14.2%) in different educational hospitals (56). 

In this review, very few documents were found regarding other countries in the Middle East Region. This might be because of the language limitation, since our search was conducted only in English documents. The review indicated that while HECs in most developed countries are well established and active, in some developing countries, even if they exist, there is a gap in their activating strategies. In some cases they are only a grand name on the list of different hospital committees and do not perform any specific tasks. 

**Functions of hospital ethics committees**

Slowther et al. stated: "The healthcare ethics committees were born out of a grass-root process in American hospitals" (57). Three domains or functions must be covered by HECs in their ordinary work. First, the HEC needs to educate its members, hospital staff, and also patients about ethical issues. The second function of an HEC is to cooperate in the development and revision of various hospital policies and guidelines to facilitate service provision by hospital personnel. The third function of a HEC is the task of ethical case analysis (58). 

The committees have a variety of responsibilities including the resolution of clinicians’ ethical issues, provision of ethical training to their members (at least one individual) and individuals from other institutions, and cooperation in the formulation of institutional policies related to clinical ethical issues. Although a committee may perform only one of the mentioned tasks, most existing committees are involved in all of them (4).

The function and constitution of these committees are different from RECs, the purpose of which is considered the ethical review of research on human subjects (4). One or more of the following three functions may ordinarily be performed by the HECs in the United States (59): (1) providing ethical consultations upon the requests from clinicians or sometimes patients and their families. The main goals of an ethics consultation in hospital ethics committees is presented in table 1; (2) Assisting in the formulation of hospital policies and guidelines by presenting the required ethical input; and (3) training the health personnel of the institution (22). 

In Norway, individual patient cases are normally debated in the HEC, not with ethics consultant teams or individual consultants, as is often the case in many other countries (27). The procedures for case discussions suggested in the HEC manual are defining the ethical problem(s), describing all facts, identifying the values and pertinent laws at risk, identifying and discussing probable solutions of the case, conclusion, and follow-up and evaluation. The manual puts more emphasis on the patient’s situation, values, and interests which are to be given a central place in the committee’s work (30). A study in Norway indicated that between 1 and 8 seminars for hospital employees during the last 2 years with, altogether, 4,400 participants had been arranged by 30 of the 31 HECs. To elaborate on the ethical guidelines, 26 of the HECs had become involved. The topics of the guidelines were end-of-life issues (including not attempting resuscitation and caring for the relatives), patient autonomy issues/involuntary treatment, prioritization issues, confidentiality, communication with patients on Facebook and cultural issues/language problems (30).

In Croatia, the implementation of ethical principles of the medical profession, approval of research activities (protocols) within the healthcare institution, supervision of drug and medical device trails, supervision of organ procurement from deceased persons, and resolution of other ethical issues in the health institution are undertaken by the ethics committees (28). Table 2 shows the suggested functions for hospital ethics committees.

**Table 1:** The main goals of ethical consultation in hospital ethics committees (9)

Developing relevant interventions to protect patient rights Proposing solutions to real or imagined conflicts Modifying patient care protocols to improve quality Enhancing patient/family satisfaction Providing the staff with ethical education Developing strategies to prevent future ethical issues Satisfying the perceived needs of the staff Providing the staff with moral support Discontinuing undesirable or inefficient treatments Minimizing the risk of legal liability

***The challenges of hospital ethics committees***

Challenges of HECs can be divided into three main categories based on the reviewed literature. 

*Clarifying and maintaining their position inside the institution*

Although HECs may have a multiplicity of goals and functions, one of the challenges that all HECs share is clarifying and maintaining their position inside their respective institution. HECs in Europe have to protect the interests of both individuals and the organization while maintaining a critical independence (8).

*Convincing professionals of the necessity of engaging patients and their families in the decision making process*

Persuading health professionals of the significance of engaging patients and their families in medical decision-making is also a challenge. In fact, when a discussion about the hospitals’ "do not resuscitate policy" was started, some committee members and other clinicians involved in the initial development of the policy disagreed with the disclosure of information and the engagement of patients and their families in "do not resuscitate" decisions. Finally, the committee agreed on a policy that ensured the patients’ right to be involved in making such decisions, unless exceptional conditions were present. Recent media publicity has supported the committee's point of view (31).

*Educational challenges*

In the educational setting, a major challenge was to convince the staff, especially the junior medical staff, of the equal significance of ethical issues and clinical teaching. In fact, medical students are more focused on acquiring factual clinical knowledge than on discussing the ethical issues they face (31). 

According to a recent national survey in the US, 81% of the 519 surveyed hospitals and 100% of hospitals with over 400 beds provided ethics consultation services. Despite these noticeable rates, there is still ongoing debate about educating bioethicists, the objectives of ethics consultation, methods of evaluating consultation outcomes, and the best approach toward ethics consultation (9).

Developing more objective methods for the evaluation of effectiveness and providing the required ethical education for the members of such committees can be considered as the future challenges in this field. The relationships between these committees and the national structure and guidelines for ethics committees in some countries, such as New Zealand, need further attention. In fact, particular requirements for ethical approval of research projects may not be relevant when individual clinical situations are concerned (4).

A multiplicity of concerns has been raised about HECs. HECs can damage the doctor-patient relationship which is a delicate and vital component of health care, decrease doctors’ professional autonomy, and gradually undermine their responsibility and authority to act in the best interest of their patients (61).

**Table 2:** Suggested functions for hospital ethics committees (60)

Holding regular monthly or quarterly educational sessions on relevant ethical issues for not only HEC members and hospital staff, but also patients Setting annual goals for the ethics committee and regular evaluation of the steps taken toward their achievement Reviewing the policies proposed by the committee Encouraging each member of the committee to hold meetings with the members of their department about ethical issues related to their scope of activity Providing patients and their families with brochures containing information about the process of treatment decision-making, advance directives, and the role and responsibilities of the HEC Holding meetings about ethical issues encountered in different hospital units with nursing managers or head nurses Arranging meetings with other local ethics committees to share experiences and activities and assess the committee’s performance accordingly Reviewing the available literature, selecting and summarizing significant articles about ethics, and providing the staff and physicians with the produced content Designing a protocol to ensure the provision of adequate pain relief for terminally ill patients Assisting other health care institutions in the establishment of their ethics committees Asking nurses, physicians, and social workers to show the committee how they share information about advance directives, terminal care decisions, or bad news with patients Summarizing hospital policies with significant ethical content and distributing the produced summary among physicians and other hospital staff Designing and implementing a bioethics week with specific daily activities for both the day and night shift personnel Providing courses in mediation or other methods of conflict resolution Asking a sociologist to help the committee create its sociogram to clarify the relationships within the committee that influence its performance Identifying health care personnel (including physicians and nurses) who were admitted to the institution as patients and ask them to share their experiences with the committee

They may either limit the patients’ freedom of choice or neglect (rather than protect) their interests by attracting attention to various competing interests, e.g., the interests of the hospital and its personnel.

By adding an extra layer to the overburdened administrative bureaucracy of hospitals, HECs may decrease the already insufficient time available for clinical care. They may also raise unnecessary moral and even political disputes (32). Finally, in an attempt to protect themselves, HECs may act (e.g., perform analyses and provide recommendations) with extreme caution (33). 

## Conclusions

According to the results, the Americas Region and European Region countries such as United States, Canada, and Netherlands have been the most prominent considering the establishment of HECs. Nevertheless, the majority of Eastern Mediterranean Region countries and South-East Asia Region countries are only beginning to establish these committees in their hospitals. The major functions of HECs were ethics consultation, education about ethical issues, and policy-making. Based on the reviewed literature, challenges of the HECs can be divided into the 3 main categories of elucidating and establishing their position within the institution, persuading professionals of the importance of participating patients and families in decision-making, and challenges related to providing education.

The authors of this study tried to present an integrated image of the development of HECs and their performance and challenges around the world. Since the inception of these committees was in Western countries, the findings of this study may be used as a guide by health policymakers and managers who are only beginning to establish these committees in their hospitals. Moreover, health service providers in countries, where there is still no report about activities of HECs or these committees do not act efficiently, do not have any information about the performance of such committees. Therefore, the results of this study help health service providers become familiar with the status of these committees in different countries and their main functions.

There is a need for further research addressing the real gaps and some of the institutional challenges of HECs and evaluating the functions of HECs. 
